# Clinical Profile, Risk Factors, and Complications in Young-Onset Type 2 Diabetes Mellitus

**DOI:** 10.7759/cureus.68497

**Published:** 2024-09-03

**Authors:** Rahul Dhadse, Dhirendra Yadav, Leena Thakur, Swati Chavan, Rupal Padhiyar, Shifa Karatela, Alhad Mulkalwar

**Affiliations:** 1 Department of Internal Medicine, Government District Hospital, Gadchiroli, IND; 2 Department of Internal Medicine, Lokmanya Tilak Municipal Medical College and General Hospital, Mumbai, IND; 3 Department of Medicine, Medical College Baroda and Shri Sayajirao General (SSG) Hospital, Vadodara, IND; 4 Department of Pharmacology, Dr. D.Y. Patil Medical College, Hospital and Research Centre, Dr. D. Y. Patil Vidyapeeth (Deemed to be University), Pune, IND

**Keywords:** young-onset diabetes mellitus, obesity, diabetic retinopathy, diabetic nephropathy (dn), diabetic peripheral neuropathy (dpn)

## Abstract

Background

Young-onset type 2 diabetes mellitus (T2DM), defined as a diagnosis before the age of 45, is an increasingly common and aggressive form of diabetes. This population is at a heightened risk of developing complications earlier in life due to longer disease duration and often suboptimal glycemic control. Complications such as diabetic neuropathy, retinopathy, and nephropathy are significant concerns, leading to reduced quality of life and increased morbidity.

Objective

To investigate the clinical profile and risk factors associated with complications of young-onset T2DM and to analyze the correlation between the age of onset and other parameters and the development of these complications.

Methods

We conducted a cross-sectional study on young-onset T2DM patients (<45 years) to investigate the prevalence and associated factors of diabetic complications. Variables analyzed included age, gender, BMI, waist-hip ratio, duration of diabetes, age at diagnosis, proteinuria, and glycosuria, along with biochemical markers such as HbA1C (glycated hemoglobin), serum cholesterol, triglycerides, and C-peptide levels.

Results

The average age of participants in our study was 34.76 ± 6.91 years. The mean BMI was 26.68 ± 3.35, with a mean cholesterol level of 169.84 ± 55.64 and a mean triglyceride level of 205.79 ± 67.49. The average HbA1c level was 9.82 ± 2.44. Diabetic neuropathy was found to increase significantly with advancing age (p < 0.001), longer duration of diabetes (p < 0.001), higher mean levels of HbA1C (p < 0.001), serum cholesterol (p = 0.006), and serum triglycerides (p = 0.010), as well as with lower levels of serum C-peptide (p = 0.025). The severity of kidney damage showed a significant association with older age (p = 0.049), longer diabetes duration (p < 0.01), elevated mean levels of HbA1C (p = 0.0002), and serum cholesterol (p = 0.0310). Diabetic retinopathy increased significantly with advancing age (p < 0.001), longer diabetes duration (p < 0.001), higher mean levels of HbA1C (p < 0.001), and serum triglycerides (p = 0.013).

Conclusion

Young-onset T2DM is associated with significant risks for neuropathy, retinopathy, and nephropathy, particularly with increasing age and longer disease duration. Higher HbA1C, serum cholesterol, and triglyceride levels are prevalent among those with complications. These findings underscore the need for early intervention and targeted management strategies to mitigate complications in this high-risk population.

## Introduction

The incidence of type 2 diabetes mellitus (T2DM) in young adults, particularly in the Indian population, is rising rapidly. Patients with young-onset T2DM (under 45 years) often have obesity, dyslipidemia, high insulin resistance, and cardiovascular risk factors such as hypertension. Globally, diabetes prevalence among adults is projected to increase from 6.4% in 2003 to 7.7% by 2025, with T2DM expected to constitute over 90% of cases. Despite their youth and relatively short duration of diabetes, individuals with young-onset T2DM are at a heightened risk for complications such as nephropathy and cardiovascular diseases (CVD). This underscores the urgent need for targeted attention due to the high prevalence and elevated risk of adverse outcomes [1].

The definition of early-onset T2DM varies, typically encompassing its development in children, adolescents, youth, and young adults. According to National Institute for Health and Care Excellence (NICE) guidelines, early-onset T2DM is defined as onset before the age of 40. For practical purposes, this can be categorized into three groups: pediatric (under 18 years), youth (18-25 years), and young adults (over 25 years). Given that cardiovascular risk and mortality are higher in those with T2DM who are under 45 years old compared to those over 45, it is advisable to focus studies on young-onset T2DM in the 18-45 age range to better understand their specific characteristics [2].

The rate of development of T2DM in less than 40 years of age has grown exponentially, especially in recent years [3-5]. This is leading to a variety of medical and societal concerns. Young patients developing T2DM are exposed to an extended duration of risk factors such as hyperglycemia over many years and are subsequently predisposed to various complications. Young-onset T2DM usually have a more aggressive disease with a high risk of complications and there are hardly any evidence-based clinical interventions or policies targeting this age either in detection or treatment. They are the most productive segment of society, and physical and psychosocial morbidities associated with young-onset T2DM will have serious consequences [6]. This will invariably create a major public problem and will have a significant impact on public healthcare policy and services.

T2DM in young has several key drivers/factors that influence its development either directly or indirectly. There is an association of maternal hyper- or hypo-nutrition with an increased risk of T2DM in adolescence and adult life [7,8]. Obesity is one of the key drivers of the development of T2DM in young due to its association with metabolic syndrome and insulin resistance [9,10]. Physical inactivity/sedentary lifestyle predisposes towards the development of metabolic syndrome and insulin resistance and subsequently to T2DM [11,12]. A family history of T2DM, along with other risk factors, can predispose young patients to diabetes. Female patients are at higher risk of T2DM especially if they have insulin resistance and concomitant polycystic ovarian syndrome [13,14]. 

T2DM in young is more aggressive and has evidence of premature microvascular and macrovascular complications [5]. They have 10 years less life expectancy compared to T2DM developing after 40 years of age [5]. A two-fold increase in mortality was noted in T2DM of young (15-30 years) compared to T1DM [15]. T2DM in young has a 20% higher risk of microalbuminuria compared to patients with T2DM in > 40 years of age [2]. They also have a high risk of premature atherosclerosis and CVD with an eightfold increased risk of any macrovascular complications and a 14-fold increased risk of myocardial infarction compared to normal controls [2]. Hence, there is an urgent need to study young-onset T2DM to fill the gaps in our understanding of its natural history due to the lack of epidemiological and randomized control trials. This will help in the formulation of strategies for prevention, early detection, and standardizing treatment of T2DM in this age group of patients. In this study, various presentations and potential risk factors have been elaborated on, which gives a wide view of the clinical profile of the disease among participants who are getting medical treatment in a tertiary care center.

## Materials and methods

A cross-sectional study was conducted at Lokmanya Tilak Medical College and Government Hospital, including outpatient department (OPD) patients and indoor medical ward patients for 18 months, following the approval of the Institutional Ethics Committee. Based on diabetic OPD and IPD (inpatient department) data, it was estimated that approximately 300 young diabetic patients (<45 years) would visit the diabetes OPD during the study period, given that around 4,000 patients attend the diabetic OPD annually, with about 5% (200) being young diabetics. Due to time constraints and the scope of the study, 100 participants were included using consecutive sampling with serial recruitment. Inclusion criteria were age 18-45 years and diagnosed with T2DM, while the exclusion criteria included type 1 DM, gestational DM, age <18 years or >45 years, and other types of DM, such as monogenic DM (MODY) and LADA (latent autoimmune diabetes in adults). Patients were diagnosed with T2DM based on the American Diabetes Association classification (ADA 2020) [1].

Complications studied included retinopathy (non-proliferative/proliferative) based on fundus examination, neuropathy (sensory and motor, mono and polyneuropathy) based on clinical assessment and nerve conduction studies (NCS) in selected patients, and nephropathy based on albuminuria and declining renal function [16]. Demographic details and detailed history, including present complaints, history, family history of DM in first-degree relatives, personal history, and co-morbidities such as hypertension, were collected.

Patients underwent a thorough examination, including vital signs, and general and systemic examinations. Indian classification of BMI and waist-hip ratio was followed [17]. Routine investigations included complete blood count (CBC), liver function test (LFT), renal function test (RFT), fasting blood sugar/postprandial blood sugar (FBS/PLBS), lipid profile, glycated hemoglobin (HbA1c) level, serum insulin level, C-peptide level, urine routine microscopy, and 24-hour urinary protein.

Data collected were entered into Microsoft Excel 2021 (Microsoft® Corp., Redmond, WA) and analyzed using the Statistical Product and Service Solutions (SPSS, version 29; IBM SPSS Statistics for Windows, Armonk, NY) software. An unpaired T-test was used to compare quantitative variables and a chi-square test was used for qualitative ones. A two-tailed p-value < 0.05 was considered significant.

## Results

Among the 100 participants, 34% were females, and 66% were males, with a male-to-female ratio of 1.94:1. Seventy-eight participants were married, and 22 were unmarried. The average age was 34.76 ± 6.91 years, with a median age of 35.5 years. The average duration of diabetes was 3.01 ± 3.29 years, with a range from newly diagnosed to 16 years and a median of two years. Detailed findings from examinations and investigations are summarized in Table 1.

**Table 1:** Examination and investigation findings of the study participants SD: Standard Deviation, IQR: Interquartile Range, BMI: Body Mass Index, BUN: Blood Urea Nitrogen, HbA1c: Glycated Hemoglobin

Parameter	Mean ± SD	Median (IQR)	Range
Age (years)	34.76 ± 6.91	35.50 (10.50)	22-45
Duration of diabetes (years)	3.01 ± 3.29	2.00 (4.69)	0-16
BMI (kg/m^2^)	26.68 ± 3.35	26.10 (4.60)	19.5-37.4
Waist hip ratio	0.86 ± 0.06	0.87 (0.07)	0.7-0.98
Haemoglobin (g/dL)	10.64 ± 2.58	10.25 (3.28)	4.5-16.2
BUN (mg/dL)	18.99 ± 15.17	14.00 (7.25)	6-89
Creatinine (mg/dL)	1.45 ± 1.41	0.90 (0.70)	0.4-9
Total bilirubin (mg/dL)	0.90 ± 0.81	0.70 (0.40)	0.3-6.4
Direct bilirubin (mg/dL)	0.55 ± 0.60	0.40 (0.30)	0.1-4.8
Total protein (g/dL)	6.52 ± 0.81	6.55 (1.10)	4.6-8.7
Albumin (g/dL)	3.49 ± 0.81	3.50 (1.03)	1.8-5.4
Fasting blood sugar (mg/dL)	236.10 ± 100.28	212.50 (144.75)	99-586
Post-lunch blood sugar (mg/dL)	299.03 ± 123.62	270.50 (175.25)	135-705
HbA1c (%)	9.82 ± 2.44	9.15 (3.63)	6.2-15
Cholesterol (mg/dL)	169.84 ± 55.64	156.00 (68.25)	87-345
Triglycerides (mg/dL)	205.79 ± 67.49	194.50 (106)	75-387
C-peptide level (ng/mL)	4.11 ± 1.30	4.20 (2.35)	1.6-6.4
S. Insulin level (mIU/L)	9.17 ± 3.43	8.60 (5.80)	3.9-18

**Figure 1:**
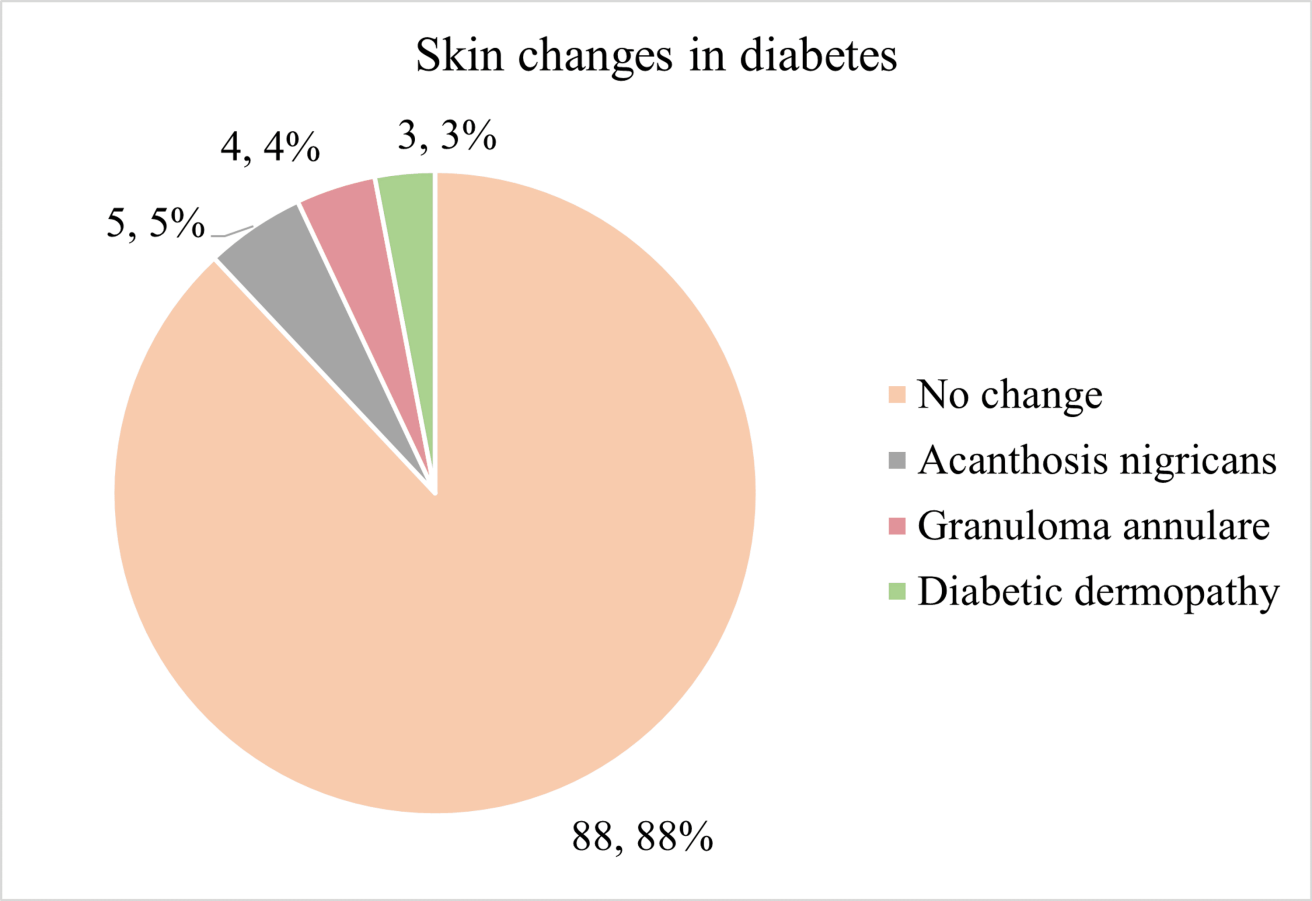
Skin changes in the study participants

A total of 37 participants (37%) reported a history of consuming addictive substances such as alcohol, smoking, and chewable tobacco. Among the 100 diabetic participants, 17% also had hypertension, and 38% had a family history of diabetes. Most participants (n = 88) showed no skin involvement, but some had diabetes-related skin conditions such as acanthosis nigricans, diabetic dermopathy, and granuloma annulare. Figure 1 shows the skin changes among study participants.

**Figure 2:**
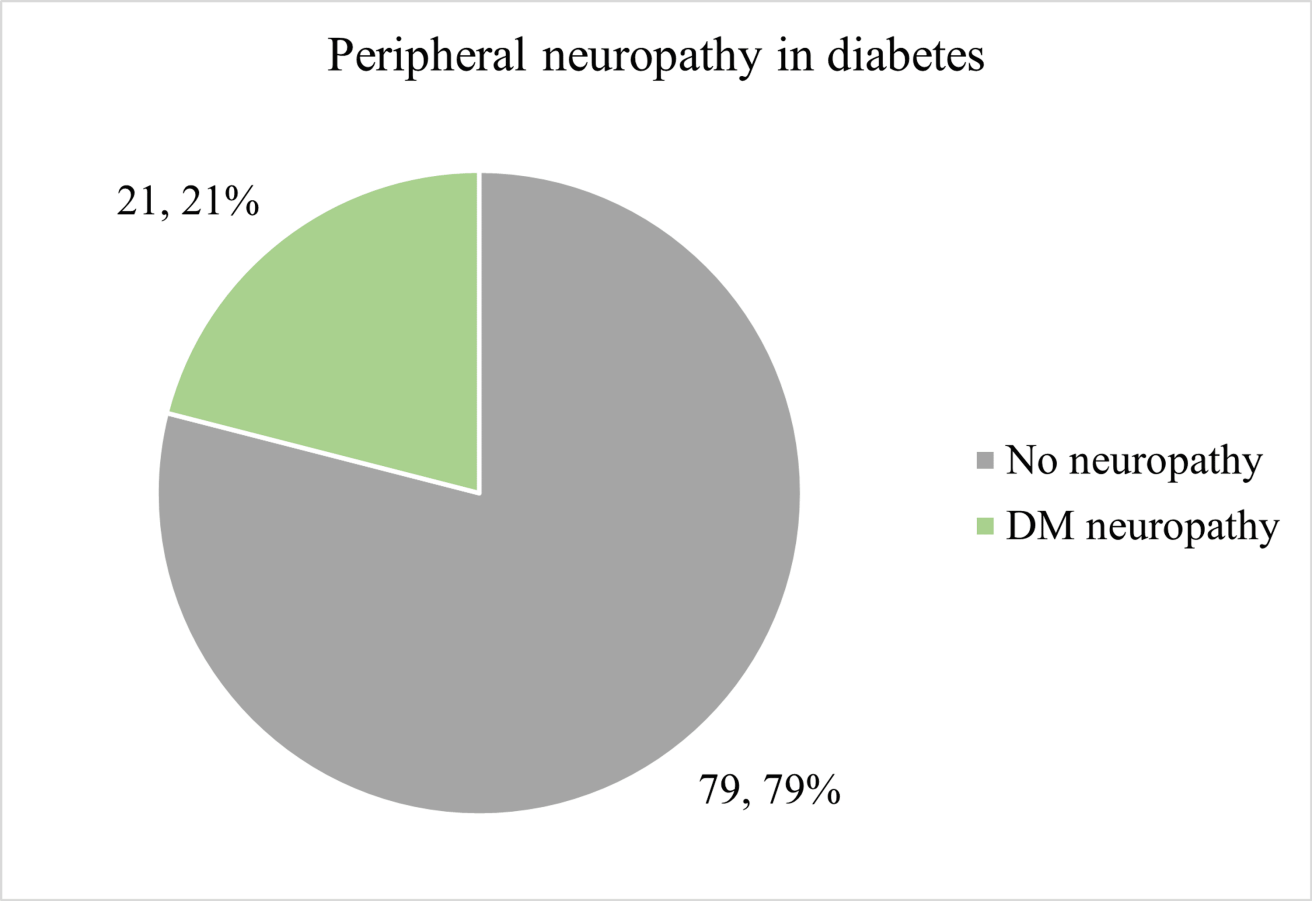
Distribution of study participants based on peripheral neuropathy according to EMG/NCV finding DM: Diabetes Mellitus, EMG/NCV: electromyography/nerve conduction velocity

Figure 2 shows the distribution of peripheral neuropathy among the participants.

As participants aged, the incidence of diabetic neuropathy increased significantly (p < 0.001). A higher proportion of females had diabetic neuropathy compared to males, but this difference was not statistically significant (p = 0.335). While diabetic neuropathy was more common among participants with higher BMI and increased waist-hip ratio (over 0.9 for males and 0.85 for females), these associations were not statistically significant (p = 0.085 and p = 0.078, respectively) [17]. A longer duration of diabetes was significantly associated with an increased incidence of neuropathy (p < 0.001). Participants diagnosed with diabetes at or after age 31 had higher neuropathy rates, though this was not statistically significant. Diabetic neuropathy was significantly more prevalent in participants with proteinuria or glycosuria (p < 0.01). Additionally, retinopathy was more frequent among those with diabetic neuropathy, showing a strong association (p < 0.01) (Table 2).

**Table 2:** Association of age, gender, BMI, waist-hip ratio, age of onset of diabetes, proteinuria, glycosuria, and fundus examination with diabetic neuropathy among participants EMG/NCV: Electromyography/nerve conduction velocity, DM: Diabetes mellitus, NAD: No abnormalities detected, PDR: Proliferative diabetic retinopathy, NPDR: Non-proliferative diabetic retinopathy Statistically significant values are bolded (p < 0.05).

Parameter	EMG/NCV, N (%)		Chi-square test
	No neuropathy	DM neuropathy	
Age			
18-25, n = 13	13 (100%)	0 (0%)	X^2^ (2, n = 100) = 13.94, p < 0.001
26-35, n = 37	34 (91.9%)	3 (8.1%)	
36-45, n = 50	32 (64%)	18 (36%)	
Gender			
Male, n = 66	54 (81.8%)	12 (18.2%)	X^2^ (1, n = 100) = 0.93, p = 0.335
Female, n = 34	25 (73.5%)	9 (26.5%)	
Duration of diabetes			
Recently diagnosed, n = 10	9 (90%)	1 (10%)	X^2^ (4, n = 100) = 34.63, p < 0.001
1-12 months, n = 26	25 (96.2%)	1 (3.8%)	
1-4 years, n = 35	32 (91.4%)	3 (8.6%)	
5-9 years, n = 25	13 (52%)	12 (48%)	
≥10 years, n = 4	0 (0%)	4 (100%)	
Onset of diabetes			
≤ 30 years, n = 42	35 (83.3%)	7 (16.7%)	X^2^ (1, n = 100) = 0.82, p = 0.365
≥ 31 years, n = 58	44 (75.9%)	14 (24.1%)	
Body mass index			
Normal, n = 10	10 (100%)	0 (0%)	X^2^ (2, n = 100) = 4.92, p = 0.085
Overweight, n = 23	20 (87%)	3 (13%)	
Obese, n = 67	49 (73.1%)	18 (26.9%)	
Waist hip ratio			
Normal, n = 64	54 (84.4%)	10 (15.6%)	X^2^ (1, n = 100) = 3.09, p = 0.078
Increased, n = 36	25 (69.4%)	11 (30.6%)	
Proteinuria			
Yes, n = 17	8 (47.1%)	9 (52.9%)	X^2^ (1, n = 100) = 12.60, p < 0.01
No, n = 83	71 (85.5%)	12 (14.5%)	
Glycosuria			
Yes, n = 45	26 (57.8%)	19 (42.2%)	X^2^ (1, n = 100) = 22.21, p < 0.01
No, n = 55	53 (96.4%)	2 (3.6%)	
Fundus for retinopathy			
NAD, n = 72	67 (93.1%)	5 (6.9%)	X^2^ (2, n = 100) = 30.78, p < 0.01
NPDR, n = 13	6 (46.2%)	7 (53.8%)	
PDR, n = 15	6 (40%)	9 (60%)	

The results of the t-test revealed that participants with neuropathy had significantly higher mean HbA1C (p = < 0.001), serum cholesterol (p = 0.006), and serum triglyceride levels (p = 0.010) than those without neuropathy. However, participants with neuropathy had significantly lower serum C-peptide levels (p = 0.025) than those without neuropathy (Table 3).

**Table 3:** Comparison of the values of HbA1C levels, serum cholesterol levels, serum triglyceride levels, and C-peptide levels among diabetics with and without neuropathy EMG/NCV: Electromyography/nerve conduction velocity, DM: Diabetes mellitus, SD: Standard deviation, SEM: Standard error of the mean, HbA1C: Glycated hemoglobin Statistically significant values are bolded (p < 0.05).

Blood parameters	EMG NCV findings		t-test	P-value
	DM neuropathy, mean ± SD (SEM)	No neuropathy, mean ± SD (SEM)		
HbA1C (%)	12.467 ± 2.002 (0.437)	9.115 ± 2.040 (0.229)	6.718	< 0.001
Cholesterol (mg/dL)	198.95 ± 54.46 (11.88)	162.10 ± 53.66 (6.04)	2.789	0.006
Triglyceride (mg/dL)	239.14 ± 69.82 (15.24)	196.92 ± 64.44 (7.25)	2.623	0.010
C-peptide level (ng/mL)	3.62 ± 1.15 (0.25)	4.24 ± 1.32 (0.15)	1.963	0.025

**Figure 3:**
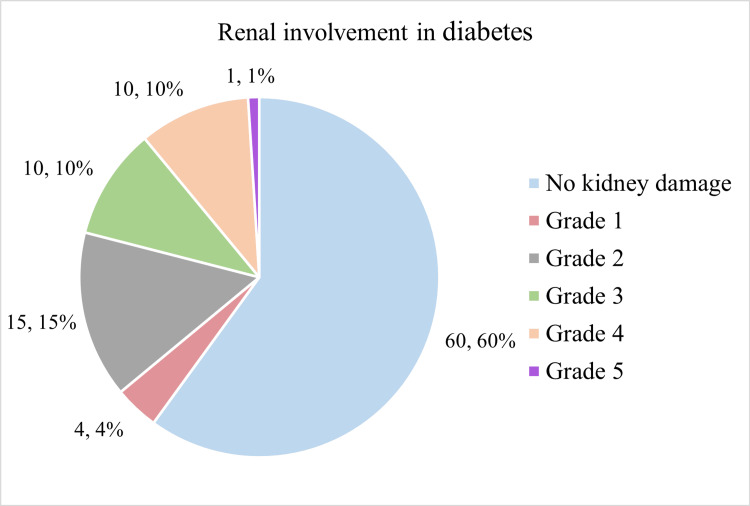
Distribution of study participants based on renal damage

Figure 3 shows the distribution of renal involvement among the participants which was done based on estimated glomerular filtration rate (eGFR) calculations [16].

Participants with higher age had increased severity of kidney damage, which was statistically significant (p = 0.049). Females had a higher incidence of severe kidney damage, though not statistically significant (p = 0.734). Increased diabetes duration was significantly linked to more severe kidney damage (p < 0.01). While older age at diabetes onset was associated with more severe kidney damage, this was not statistically significant (p = 0.224). No significant association was found between BMI or waist-hip ratio and kidney damage (p = 0.73 and p = 0.41, respectively). More patients with kidney involvement had proteinuria and glycosuria, with both showing statistically significant associations (p < 0.001 and p = 0.001) (Table 4).

**Table 4:** Association of age, gender, BMI, waist-hip ratio, age of onset of diabetes, proteinuria, and glycosuria with diabetic nephropathy among participants NAD: No abnormalities detected, BMI: Body mass index Statistically significant values are bolded (p < 0.05).

Parameter	Kidney Involvement, N (%)						Chi-square test
	NAD	Grade 1	Grade 2	Grade 3	Grade 4	Grade 5	
Age							
18 - 25, n = 13	10 (76.9%)	1 (7.7%)	1 (7.7%)	1 (7.7%)	0 (0%)	0 (0%)	X^2^ (10, n = 100) = 18.33, p = 0.049
26 - 35, n = 37	28 (75.7%)	1 (2.7%)	3 (8.1%)	0 (0%)	4 (10.8%)	1 (2.7%)	
36 - 45, n = 50	22 (44%)	2 (4%)	11 (22%)	9 (18%)	6 (12%)	0 (0%)	
Gender							
Male, n = 66	41 (62.1%)	2 (3%)	10 (15.2%)	7 (10.6%)	6 (9.1%)	0 (0%)	X^2^ (5, n = 100) = 2.78, p = 0.734
Female, n = 34	19 (55.9%)	2 (5.9%)	5 (14.7%)	3 (8.8%)	4 (11.8%)	1 (2.9%)	
Duration of diabetes							
Recently diagnosed, n = 10	7 (70%)	0 (0%)	0 (0%)	2 (20%)	0 (0%)	1 (10%)	X^2^ (20, n = 100) = 44.90, p = 0.001
1 - 12 months, n = 26	20 (76.9%)	2 (7.7%)	2 (7.7%)	1 (3.8%)	1 (3.8%)	0 (0%)	
1 - 4 years, n = 35	25 (71.4%)	2 (5.7%)	4 (11.4%)	2 (5.7%)	2 (5.7%)	0 (0%)	
5 - 9 years, n = 25	8 (32%)	0 (0%)	8 (32%)	3 (12%)	6 (24%)	0 (0%)	
≥10 years, n = 4	0 (0%)	0 (0%)	1 (25%)	2 (50%)	1 (25%)	0 (0%)	
Onset of diabetes							
≤ 30 years, n = 42	31 (73.8%)	1 (2.4%)	4 (9.5%)	2 (4.8%)	4 (9.5%)	0 (0%)	X^2^ (5, n = 100) = 6.95, p = 0.224
≥ 31 years, n = 58	29 (50%)	3 (5.2%)	11 (19%)	8 (13.8%)	6 (10.3%)	1 (1.7%)	
Body mass index							
Normal, n = 10	6 (60%)	1 (10%)	1 (10%)	1 (10%)	1 (10%)	0 (0%)	X^2^ (10, n = 100) = 6.99, p = 0.73
Overweight, n = 23	15 (65.2%)	1 (4.3%)	2 (8.7%)	3 (13%)	1 (4.3%)	1 (4.3%)	
Obese, n = 67	39 (58.2%)	2 (3%)	12 (17.9%)	6 (9%)	8 (11.9%)	0 (0%)	
Waist hip ratio							
Normal, n = 64	39 (60.9%)	4 (6.3%)	7 (10.9%)	6 (9.4%)	7 (10.9%)	1 (1.6%)	X^2^ (5, n = 100) = 5.02, p = 0.41
Increased, n = 36	21 (58.3%)	0 (0%)	8 (22.2%)	4 (11.1%)	3 (8.3%)	0 (0%)	
Proteinuria							
Yes, n = 17	0 (0%)	4 (23.5%)	3 (17.6%)	5 (29.4%)	5 (29.4%)	0 (0%)	X^2^ (5, n = 100) = 52.78, p < 0.001
No, n = 83	60 (72.3%)	0 (0%)	12 (14.5%)	5 (6%)	5 (6%)	1 (1.2%)	
Glycosuria							
Yes, n = 45	19 (42.2%)	4 (8.9%)	6 (13.3%)	8 (17.8%)	8 (17.8%)	0 (0%)	X^2^ (5, n = 100) = 20.07, p = 0.001
No, n = 55	41 (74.5%)	0 (0%)	9 (16.4%)	2 (3.6%)	2 (3.6%)	1 (1.8%)	

Participants with nephropathy had higher mean HbA1C and serum cholesterol levels, with statistically significant differences (p = 0.0002 and p = 0.0310, respectively). They also had higher mean serum triglyceride levels, but this difference was not statistically significant (p = 0.1064). Interestingly, participants with nephropathy had lower mean serum C-peptide levels, though this difference was also not statistically significant (p = 0.1280) (Table 5).

**Table 5:** Comparison and association of values of HbA1C, serum cholesterol, serum triglyceride, and C-peptide levels among diabetics with and without kidney involvement DM: Diabetes mellitus, SD: Standard deviation, SEM: Standard error of the mean, HbA1C: Glycated hemoglobin Statistically significant values are bolded (p < 0.05).

Blood Parameters	Kidney involvement		t-test	P-value
	Renal damage, mean ± SD (SEM)	No renal damage, mean ± SD (SEM)		
HbA1C (%)	10.90 ± 2.54 (0.40)	9.09 ± 2.10 (0.27)	3.867	0.0002
Cholesterol (mg/dL)	184.48 ± 54.13 (8.56)	162.08 ± 54.91 (7.09)	2.188	0.0310
Triglyceride (mg/dL)	219.15 ± 65.12 (10.30)	196.88 ± 68.10 (8.79)	1.630	0.1064
C-peptide level (ng/mL)	3.86± 1.32 (0.21)	4.27 ± 1.27 (0.16)	1.535	0.1280

**Figure 4:**
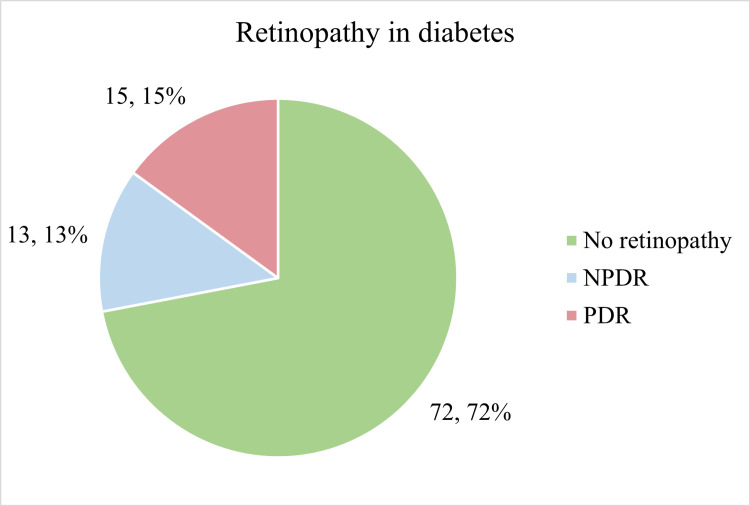
Distribution of study participants based on retinopathy PDR: Proliferative diabetic retinopathy, NPDR: Non-proliferative diabetic retinopathy

Figure 4 shows the distribution of retinopathy among the participants.

As participants aged, the incidence of diabetic retinopathy increased significantly (p < 0.001). No association was found between gender and retinopathy (p = 0.61). Although diabetic retinopathy was more common among participants with higher BMI and increased waist-hip ratio (over 0.9 for males and 0.85 for females), these associations were not statistically significant (p = 0.13 and p = 0.073, respectively). A longer duration of diabetes was significantly associated with an increased incidence of retinopathy (p < 0.001). Participants diagnosed with diabetes at or after age 31 had higher neuropathy rates (p = 0.04). Diabetic neuropathy was significantly more prevalent in participants with proteinuria (p < 0.001) or glycosuria (p < 0.01) (Table 6).

**Table 6:** Association of age, gender, BMI, waist-hip ratio, age of onset of diabetes, proteinuria, and glycosuria with diabetic retinopathy among participants PDR: Proliferative diabetic retinopathy, NPDR: Non-proliferative diabetic retinopathy Statistically significant values are bolded (p < 0.05).

Parameter	Fundus examination for retinopathy, N (%)			Chi-square test
	No retinopathy	NPDR	PDR	
Age				
18-25, n = 13	13 (100%)	0 (0%)	0 (0%)	X^2^ (4, n = 100) = 21.28, p < 0.001
26-35, n = 37	33 (89.2%)	3 (8.1%)	1 (2.7%)	
36-45, n = 50	26 (52%)	10 (20%)	14 (28%)	
Gender				
Male, n = 66	49 (74.2%)	7 (10.6%)	10 (15.2%)	X^2^ (2, n = 100) = 0.99, p = 0.61
Female, n = 34	23 (67.6%)	6 (17.6%)	5 (14.7%)	
Duration of diabetes				
Recently diagnosed, n = 10	7 (70%)	1 (10%)	2 (20%)	X^2^ (8, n = 100) = 39.23, p < 0.001
1-12 months, n = 26	25 (96.2%)	1 (3.8%)	0 (0%)	
1-4 years, n = 35	31 (88.6%)	2 (5.7%)	2 (5.7%)	
5-9 years, n = 25	9 (36%)	7 (28%)	9 (36%)	
≥10 years, n = 4	0 (0%)	2 (50%)	2 (50%)	
Onset of diabetes				
≤ 30 years, n = 42	35 (83.3%)	5 (11.9%)	2 (4.8%)	X^2^ (2, n = 100) = 6.41, p = 0.04
≥ 31 years, n = 58	37 (63.8%)	8 (13.8%)	13 (22.4%)	
Body mass index				
Normal, n = 10	9 (90%)	1 (10%)	0 (0%)	X^2^ (4, n = 100) = 7.11, p = 0.13
Overweight, n = 23	20 (87%)	2 (8.7%)	1 (4.3%)	
Obese, n = 67	43 (64.2%)	10 (14.9%)	14 (20.9%)	
Waist hip ratio				
Normal, n = 64	51 (79.7%)	6 (9.4%)	7 (10.9%)	X^2^ (2, n = 100) = 5.21, p = 0.073
Increased, n = 36	21 (58.3%)	7 (19.4%)	8 (22.2%)	
Proteinuria				
Yes, n = 17	5 (29.4%)	7 (41.2%)	5 (29.4%)	X^2^ (2, n = 100) = 20.5, p < 0.001
No, n = 83	67 (80.7%)	6 (7.2%)	10 (12.0%)	
Glycosuria				
Yes, n = 45	23 (51.1%)	11 (24.4%)	11 (24.4%)	X^2^ (2, n = 100) = 18.06, p < 0.01
No, n = 55	49 (89.1%)	2 (3.6%)	4 (7.3%)	

Participants with retinopathy had significantly higher mean HbA1C (p < 0.001), serum cholesterol (p = 0.007), and serum triglyceride levels (p = 0.013). However, there was no statistically significant difference in mean serum C-peptide levels, with lower mean levels in those with retinopathy (Table 7).

**Table 7:** Comparison of values of HbA1C, serum cholesterol, serum triglyceride, and C-peptide levels among diabetics with and without retinopathy DM: Diabetes mellitus, SD: Standard deviation, SEM: Standard error of the mean, HbA1C: Glycated hemoglobin Statistically significant values are bolded (p < 0.05).

Blood parameters	Fundus examination		t-test	P-value
	DM Retinopathy, mean ± SD (SEM)	No retinopathy, mean ± SD (SEM)		
HbA1C (%)	12.08 ± 2.41 (0.45)	8.94 ± 1.82 (0.21)	7.508	< 0.001
Cholesterol (mg/dL)	193.43 ± 54.13 (10.23)	160.67 ± 53.82 (6.34)	2.729	0.007
Triglyceride (mg/dL)	232.43 ± 61.26 (11.58)	195.43 ± 67.34 (7.94)	2.528	0.013
C-peptide level (ng/mL)	3.75 ± 1.21 (0.23)	4.25 ± 1.32 (0.16)	1.734	0.086

## Discussion

Young-onset T2DM is a modern menace that has arisen due to the combination of poor lifestyle choices, sedentary habits, diet changes, and genetic predispositions. In our study, the average BMI of the participants was 26.68 ± 3.35, which is classified as obese [17]. The mean cholesterol level was 169.84 ± 55.64, placing it in the high-risk category, and the mean triglyceride level was 205.79 ± 67.49, which was also considered high, as per the Indian Heart Association [18]. The average HbA1c was 9.82 ± 2.44, which is diagnostic of diabetes and indicates poor glycaemic control. Hence, dyslipidemia, obesity, and poor glycaemic control were identified as significant risk factors for young-onset T2DM.

The proportion of males (66, 66.0%) was found to be higher than females (34, 34.0%) in our study. There was no significant association found in terms of gender and young-onset T2DM, as seen in work done by Wong et al. [19] and Luk et al. [20]. The mean age of the participants in the current study was 34.76 ± 6.91 years, with a median age of 35.5 years. Hillier et al. have supported this age group inclusion, as the cardiovascular risk and mortality are higher in T2DM <45 years of age compared to T2DM >45 years of age. Their team found it appropriate to include young-onset T2DM between 18-45 years of age for studying their characteristics at an early point to increase the likelihood of successful interventions in reducing the frequency of complications among the participants [2].

Among the 100 participants, there were 17 with a positive history of alcohol consumption, 17 with a history of smoking, and eight with a history of using smokeless tobacco. A total of 63 participants (63.0%) did not give a history of consuming any addictive substance. Work done by Ahola-Olli et al. did not find a significant association between the consumption of addictive substances and young-onset T2DM in assessing the odds ratio among participants in their prospective study [21]. A meta-analysis of 32 genome-wide association studies, involving 898,130 individuals of European ancestry, revealed genetic evidence supporting a causal link between smoking initiation and type 2 diabetes. Yuan et al. emphasized that reducing cigarette smoking initiation should be strongly recommended as a preventive measure for type 2 diabetes [22].

Lascar et al. highlighted that young-onset T2DM disproportionately affects working-age individuals, amplifying its societal impact. The study also found that this early-onset form of diabetes often presents with a more aggressive disease phenotype, leading to early complications, reduced quality of life, and poor long-term outcomes, potentially contributing to a future public health crisis [23].

Diabetic neuropathy

This study examined diabetic neuropathy in young-onset T2DM patients. Among the 100 diabetic participants, the majority of participants were found to have normal findings. However, 21 (21.0%) were found to have a detectable form of diabetic neuropathy on EMG and nerve conduction velocity tests.

The incidence increased significantly with age (p < 0.001) and longer diabetes duration (p < 0.001). Although more common in females, with higher BMI, and increased waist-hip ratio, these associations were not significant. Neuropathy was significantly linked to proteinuria, glycosuria (p < 0.01), and retinopathy (p < 0.01). Patients with neuropathy had higher HbA1C (p < 0.001), serum cholesterol (p = 0.006), and triglycerides (p = 0.010), but lower serum C-peptide levels (p = 0.025).

A study by Mao et al. found that age is an independent risk factor for the development of diabetic neuropathy in T2DM patients. It is significantly associated with both small and large nerve dysfunction, regardless of other risk factors [24].

A meta-analysis by Liu et al. found that diabetes duration, age, HbA1c levels, and the presence of diabetic retinopathy are significantly associated with increased risks of diabetic peripheral neuropathy (DPN) in diabetic patients. However, BMI, smoking, total triglycerides, and total cholesterol were not associated with increased DPN risk [25].

Diabetic nephropathy

The majority of the participants (64, 64.0%) had stage 1 kidney disease based on eGFR calculations. There were 15 participants (15.0%) who had stage 2 kidney disease and 10 participants with stage 3 and stage 4, respectively (10.0% each). Only one participant showed evidence of stage 5 kidney disease based on eGFR calculations. The use of eGFR was found to be a more objective scoring system to quantify the extent of kidney damage among participants with diabetes mellitus. Although kidney damage was identifiable on ultrasonography as well as grade 1, and grade 2 renal disease, these findings are more subjective and hence not preferred.

The majority of participants (11, 84.6%) belonging to the age group 18-25 years had stage 1 kidney disease, while there were none with stage 4 and stage 5 kidney disease. As the age groups increased, the proportion of persons having stage 1 kidney disease decreased. In contrast, the proportion of participants who were found to have higher stages of kidney disease increased. Higher age (p = 0.049) and longer diabetes duration (p < 0.01) significantly increased kidney damage. Female gender, older age at onset, BMI, and waist-hip ratio were not significant factors. Proteinuria and glycosuria were strongly linked to kidney damage (p < 0.001 and p = 0.001). Patients with nephropathy had higher HbA1C (p = 0.0002) and serum cholesterol (p = 0.0310), but triglycerides and C-peptide levels were not significant.

Yokoyama et al. studied diabetic nephropathy incidence over 25 years at a Japanese diabetes center, finding that type 2 diabetes patients with 30 years of post-pubertal diabetes had a significantly higher cumulative nephropathy incidence compared to type 1 diabetes patients. The incidence of nephropathy in type 1 diabetes has decreased over the past two decades, while it has remained stable in type 2 diabetes [26].

A study by Zheng et al. found that patients with early-onset type 2 diabetes (diagnosed before age 40) had a 3.58-fold increased risk of end-stage renal disease compared to those with late-onset diabetes, even after adjusting for various factors. Early-onset diabetes was also associated with longer disease duration, higher BMI, and worse lipid profiles [27].

Hillier et al. compared the macrovascular outcomes of diabetes by matching onset groups by age and sex with control subjects without diabetes. The study found that microalbuminuria was more common in those with early-onset type 2 diabetes compared to those with usual-onset type 2 diabetes [2].

Luk et al. conducted a prospective cohort study of Chinese patients with young-onset diabetes to investigate the incidence of renal complications. The study found that the overweight type 2 diabetes group had a significantly higher risk of progressing to end-stage renal disease, even after adjusting for age, sex, and disease duration [20].

Diabetic retinopathy

This study assessed diabetic retinopathy in young-onset type 2 diabetes patients. Additionally, 28% of study participants had some form of retinopathy present. Incidence increased significantly with age (p < 0.001) and longer diabetes duration (p < 0.001). Gender was not associated with retinopathy (p = 0.61). Higher BMI and increased waist-hip ratio were more common but insignificant factors (p = 0.13 and p = 0.073). Proteinuria (p < 0.001) and glycosuria (p < 0.01) were significantly linked to retinopathy. Participants with retinopathy had significantly higher HbA1C (p < 0.001), serum cholesterol (p = 0.007), and triglyceride levels (p = 0.013). Serum C-peptide levels were lower but not significantly different.

A study by Rajalakshmi et al. revealed that 52.7% of Asian Indian participants with early-onset T2DM exhibited some form of diabetic retinopathy. Additionally, higher glycated hemoglobin levels and lower stimulated C-peptide levels were associated with diabetic retinopathy in young individuals with T2DM [28].

Lv et al. conducted research to determine the high-risk factors associated with proliferative diabetic retinopathy (PDR). Their findings revealed that patients with early-onset T2DM had a substantially higher prevalence of PDR compared to those with late-onset T2DM [29].

Limitations

In this study, the population was drawn from a particular tertiary care hospital within a specific geographical region, so the findings cannot be generalized to the entire population. Additionally, long-term complications could not be assessed, as the study spanned only 18 months. Clinically significant macular edema (CSME) for retinopathy and microalbuminuria for nephropathy could not be considered due to the study's scope.

## Conclusions

Our study explores the significant association between young-onset T2DM and the increased risk of complications such as neuropathy, nephropathy, and retinopathy. Specifically, advancing age, longer duration of diabetes, higher HbA1c levels, elevated serum cholesterol, and increased serum triglycerides are key risk factors for these complications. The findings highlight the critical importance of early and aggressive management of glycemic control, lipid levels, and comprehensive monitoring to mitigate the risk of these complications. Tailored treatment strategies are essential to improve long-term outcomes and the quality of life for young diabetic patients, emphasizing the need for proactive and individualized care in this high-risk population.
